# Influence of dipeptidyl peptidase-4 inhibitors on glycemic variability in patients with type 2 diabetes: A meta-analysis of randomized controlled trials

**DOI:** 10.3389/fendo.2022.935039

**Published:** 2022-08-09

**Authors:** Shangyu Chai, Ruya Zhang, Ye Zhang, Richard David Carr, Yiman Zheng, Swapnil Rajpathak, Miao Yu

**Affiliations:** ^1^ Merck Research Laboratories (MRL) Global Medical Affairs, Merck Sharp & Dohme (MSD) China, Shanghai, China; ^2^ Hatter Cardiovascular Institute, University College London, UK and Ulster University, Coleraine, United Kingdom; ^3^ Merck & Co., Inc., Rahway, NJ, United States; ^4^ Department of Endocrinology, Key Laboratory of Endocrinology, National Health Commission, Peking Union Medical College Hospital, Peking Union Medical College and Chinese Academy of Medical Sciences, Beijing, China

**Keywords:** dipeptidyl peptidase-4 inhibitor, glycemic variability, mean amplitude of glycemic excursion, type 2 diabetes mellitus, meta-analysis

## Abstract

**Objective:**

The influence of dipeptidyl peptidase-4 (DPP4) inhibitors on glycemic variability compared to other oral antidiabetic drugs (OADs), measured based on the mean amplitude of glycemic excursions (MAGE), has not been comprehensively analyzed. The aim of the study was to perform a meta-analysis to compare the effects of DPP4 inhibitors on MAGE with other OADs in type 2 diabetes mellitus (T2DM) patients without concurrent insulin treatments.

**Methods:**

The Medline (PubMed), Embase (Ovid), and CENTER (Cochrane Library) databases were searched for relevant randomized controlled trials (RCTs). Study characteristics and outcome data were independently extracted by two authors. A random-effect model was used to combine the results.

**Results:**

Fourteen studies with 855 patients were included. Compared to other OADs, DPP4 inhibitors significantly reduced MAGE (mean difference [MD]: -0.69 mmol/L, 95% confidence interval [CI]: -0.95 to -0.43, P<0.001) with mild heterogeneity (I^2 =^ 28%). Predefined subgroup analyses suggested that DPP4 inhibitors were more effective in reducing MAGE compared to insulin secretagogues (MD: -0.92 mmol/L, P<0.001) and non-secretagogues (MD: -0.43 mmol/L, P=0.02), as well as compared to sulfonylureas (MD: -0.91 mmol/L, P<0.001) and sodium glucose cotransporter 2 inhibitors (MD: -0.67 mmol/L, P=0.03).

**Conclusions:**

DPP4 inhibitors may significantly reduce glycemic variability compared to other oral anti-diabetic drugs, as evidenced by MAGE in T2DM patients with no concurrent insulin treatment.

**Systematic review registration:**

INPLASY, registration number: INPLASY2021120113.

## Introduction

Vascular complications are considered the main determinants for increased disability and mortality in patients with type 2 diabetes mellitus (T2DM) (1, 2). The major cause of vascular complication in T2DM patients is poor glycemic control and long-lasting hyperglycemia (3, 4). Interestingly, subsequent evidence showed that glycemic variability, which refers to multiple fluctuations of glycemia that occur throughout the day or over longer periods of time (5), may be another key factor that may accelerate the development of diabetic complications besides persistent hyperglycemia (6, 7). Indeed, epidemiological studies have suggested that increased glycemic variability is independently associated with the development of retinopathy, nephropathy, and possibly cardiovascular events and mortality in patients with T2DM (8–10). Experimental studies also confirmed that increased glycemic variability is associated with overactive oxidative stress and inflammatory responses (11, 12), which have been implicated in vascular complications in T2DM. Therefore, glycemic variability has been established as a novel treatment target for patients with T2DM.

Among the frequently used oral antidiabetic drugs (OADs), dipeptidyl-peptidase 4 (DPP4) inhibitors have shown promising therapeutic efficacy for T2DM patients on the basis of numerous benefits besides hypoglycemic efficacy such as the preservation of islet β-cell function (13), improvement of glycemic durability (14), and suppression of abnormal hyperglucagonaemia (15). Moreover, since increased glycemic variability is correlated with poor islet β-cell function (16), a beneficial effect of DPP4-inhibitors on glycemic variability in T2DM patients has also been suggested in view of their beneficial effects on β-cell function (17). More importantly, the pharmacological mechanisms of DPP4 inhibitors involve enhancing incretin preservation, such as glucagon-like peptide-1 (GLP-1) and glucose-dependent insulinotropic polypeptide (GIP), thereby exerting their hypoglycemic effects *via* glucose-dependent insulin secretion and inhibiting glucagon production without increasing the risk of hypoglycemia (18, 19).

Conventionally, glycemic variability is evaluated based on the mean amplitude of glycemic excursions (MAGEs) *via* continuous glucose monitoring (CGM) or flash glucose monitoring (FGM) (20) and is also considered a reliable parameter for evaluating short-term within-day glycemic variability. Although some small-scale randomized controlled trials (RCTs) have been performed to compare the influences of DPP4 inhibitors and other OADs on MAGE in T2DM patients (21–34), the results of these RCTs were not consistent and a consensus on the efficacy of DPP4 inhibitors on glycemic variability compared to other OADs has not been fully determined. A previous meta-analysis (35) including only seven RCTs before 2018 showed that DPP4 inhibitors may be more effective than other OADs in reducing MAGE. However, some recently published eligible studies (30–34) have not been included in a meta-analysis. Accordingly, in this study, we aimed to perform an updated meta-analysis to summarize the current knowledge regarding the influence of DPP4 inhibitors on glycemic variability in comparison with other OADs. With more studies included, we also explored the potential influences of the study characteristics on the outcomes.

## Methods

The Preferred Reporting Items for Systematic Reviews and Meta-Analyses (PRISMA) statement (36) and the Cochrane Handbook guidelines (37) were followed during the design and implementation of the study.

### Data sources and searches

Medline (PubMed), Embase (Ovid), and CENTER (Cochrane Library) databases were searched for relevant studies with the following keywords: (1) “DPP4” OR “DPP-4” OR “dipeptidyl peptidase-4 inhibitors” OR “sitagliptin” OR “vildagliptin” OR “linagliptin” OR “saxagliptin” OR “alogliptin” OR “dutogliptin” OR “aemigliptin” OR “anagliptin” OR “teneligliptin” OR “trelagliptin” OR “omarigliptin” OR “gemigliptin” OR “evogliptin”; (2) “continuous glucose monitoring” OR “glycemic variability” OR “glyceamic variability” OR “glucose variability” OR “glucose fluctuation” OR “glycemic fluctuation” OR “mean amplitude of glycemic excursion” OR “MAGE” OR “standard deviation” OR “SD” OR “SDBG” OR “largest amplitude of glycemic excursion” OR “LAGE” OR “Coefficient of variation” OR “CV”; and (3) “random” OR “randomized” OR “randomised” OR “randomly”. Only clinical studies were considered. The references of related reviews and original articles were also searched as a complement. The final database search was conducted on July 23, 2021.

### Study selection

Studies that fulfilled the following criteria were included: (1) full-length articles published in English; (2) designed as parallel-group or crossover RCTs; (3) included adult patients with T2DM who were not treated with insulin; (4) patients were allocated to a treatment group with DPP4 inhibitors or a control group with other OADs; and (5) reported the between-group difference of changes in MAGE using CGM or FGM from the baseline for patients in each arm of the study. Studies with drug-naïve patients or T2DM patients on background OAD therapy were included. Studies including T2DM patients with concurrent insulin therapy were excluded because the influence of insulin on blood glucose fluctuations is related to various factors such as dosage and categories, which may conceal the effect of combined OADs on blood glucose fluctuations. We did not consider studies including patients treated with single-dose/single-day DPP4 inhibitors because we did not plan to evaluate the acute effect of DPP4 inhibitors on glycemic variability. In addition, non-randomized studies, studies with non-T2DM patients, and those without MAGE measurement using CGM or FGM were excluded.

### Data extraction and quality assessment

Database search, data extraction, and quality evaluation were conducted by two independent authors. Disagreements were resolved by discussion with the corresponding author. We extracted data regarding the study information (first author, publication year, and study country), study design (blind or open-label, crossover or parallel group), patient information (number of patients, mean age, sex, baseline HbA1c, and T2DM duration), details of background antidiabetic treatments, drugs and doses of DPP4 inhibitors and control OADs, treatment durations, and methods used for MAGE measurement. Quality evaluation was achieved using Cochrane’s Risk of Bias Tool (37) according to the following aspects: (1) random sequence generation; (2) allocation concealment; (3) blinding of participants and personnel; (4) blinding of outcome assessors; (5) incomplete outcome data; (6) selective outcome reporting; and (7) other potential biases.

### Data synthesis and analysis

The influences of DPP4 inhibitors on MAGE compared to controls in T2DM patients were presented as the mean difference (MD) and 95% confidence interval (CI). We used Cochrane’s Q test to detect the heterogeneity (38). The I^2^ statistic was also calculated, and an I^2^ > 50% reflected significant heterogeneity (37). Pooled analyses were calculated using a random-effect model because this method incorporates the influence of potential heterogeneity and provides a more generalized result (37). Sensitivity analysis by excluding one study at a time was used to evaluate the influence of each study on the pooled results of the meta-analysis (37). Predefined subgroup analyses were performed to evaluate the potential influences of study characteristics on the outcome. Particularly, subgroup analysis was performed to explore the relative efficacy of DPP4 inhibitors on MAGE as compared with insulin secretagogues (sulphonylureas [SUs] and glinides) and non-secretagogues, as well as individual classes of OADs. Publication bias was evaluated by visual inspection of the funnel plots and Egger’s regression asymmetry test results (39). P values < 0.05 were considered statistically significant. The RevMan (Version 5.1; Cochrane, Oxford, UK) software was applied for statistical analyses.

## Results

### Search results

**Figure 1:**
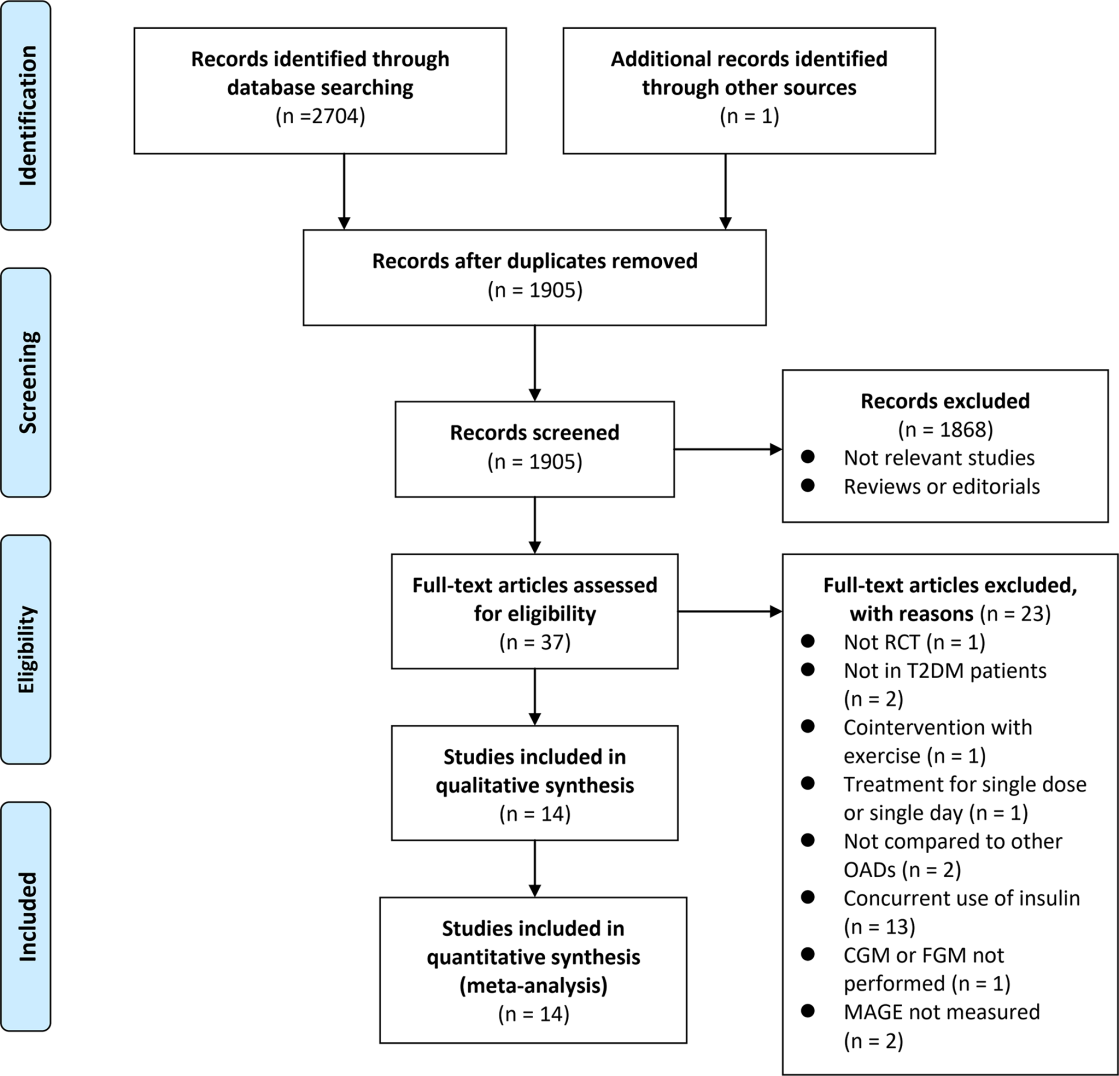
Flowchart of literature search.

The process of database search and study identification is shown in **Figure 1**. Briefly, 2,705 articles were obtained through the database search, and 1,905 were retrieved after the exclusion of duplicate records. Among them, 1,868 articles were subsequently excluded based on the titles and abstracts primarily because these studies were irrelevant to the aim of the meta-analysis. Of the 37 articles that underwent full-text review, 23 were further excluded for the reasons presented in **Figure 1**. Finally, 14 RCTs (21–34) were included.

### Study characteristics and data quality

**Table 1** shows the characteristics of the included studies. Overall, 14 RCTs comparing the effects of DPP4 inhibitors and other OADs on MAGE in T2DM patients were included (21–34). Since one study included two interventional groups with gemigliptin and sitagliptin, the two datasets were included in the meta-analysis independently. The studies included in the meta-analysis were published between 2013 and 2020. Three of them were crossover studies (21, 23, 32), while the others were parallel-group RCTs (22, 24–31, 33, 34). Various DPP4 inhibitors were used in these studies, such as vildagliptin, sitagliptin, linagliptin, and gemigliptin, and the control OADs included SUs, glinides, voglibose, pioglitazone, metformin, and dapagliflozin. The treatment durations varied between 5 and 168 days, and MAGE was measured using CGM or FGM for 24, 48, and 72 hours. The detailed quality evaluation for these studies is shown in **Table 2**, suggesting moderate study quality.

**Table 1:** Characteristics of the included RCTs.

Study	Country	Design	Patient number	Mean age	Male	Baseline HbA1c		T2DM duration	Background treatment	Intervention	Control	Treatment duration	MAGE measuring
				year	%	%		years				days	
(21)	USA	R, OL, CO	24	58.3	79.2	7.6		7	Metformin	Vildagliptin 50mg Bid	Glimepiride 2mg Qd	5	24h-CGM
(22)	Korea	R, DB	33	57.6	57.6	7.2		5.4	Metformin	Sitagliptin 100mg Qd	Glimepiride 2mg Qd	28	72h-CGM
(23)	Japan	R, CO	11	58	NR	7.3		8	Drug naïve	Sitagliptin 50mg Qd	Mitiglinide 10mg Tid	28	24h-CGM
(24)	Japan	R, OL	29	64.4	79.3	7		NR	Acarbose	Sitagliptin 50mg Qd	Mitiglinide 5-10mg Tid	28	72h-CGM
(25)	China	R	41	68.8	56.1	7.2		0	Metformin	Sitagliptin 100mg Qd	Glimepiride 1-4mg Qd	168	72h-CGM
(29)	Japan	R, SB	99	61.4	40.4	7.4		NR	Drug naïve	Linagliptin 5mg Qd	Voglibose 0.2mg Tid	84	24h-CGM
(28)	Korea	R, SB	31	49.4	71	9.6		0.8	Drug naïve	Gemigliptin 50 mg Qd	Glimepiride 2mg Qd	84	72h-CGM
(28)	Korea	R, SB	27	50.5	75	9.3		1.6	Drug naïve	Sitagliptin 100 mg Qd	Glimepiride 2mg Qd	84	72h-CGM
(22)	Korea	R, OL	25	56.4	52	7.3		NR	Metformin	Vildagliptin 50mg Bid	Pioglitazone 15mg Qd	112	72h-CGM
(22)	Korea	R, OL	34	56	58.8	7.6		6.1	Metformin	Vildagliptin 50mg Bid	Glimepiride 1-2mg Qd	84	48-h CGM
30)	Japan	R, OL	52	59.8	98.1	7.8		8.1	Drug naïve	Sitagliptin 50mg Qd	Glibenclamide 2.5 mg Qd	14	24h-CGM
(31)	Brazil	R, SB	37	61.9	0	7.3		NR	Metformin	Vildagliptin 50mg Bid	Gliclazide 60-120mg Qd	168	48-h CGM
(32)	Japan	R, CO	11	51.9	72.7	7.6		4.9	Metformin (750mg/d)	Linagliptin 5mg Qd	Metformin 500mg Tid	28	24h-CGM
(33)	Japan	R, SB	331	58.1	60.1	7.8		5.8	Drug naïve or metformin	Sitagliptin 50-100mg Qd	Dapagliflozin 5-10mg Qd	168	24h-FGM
(34)	Korea	R, SB	70	52	65.7		7.9	2.8	Drug naïve or metformin	Gemigliptin 50 mg Qd	Dapagliflozin 10mg Qd	84	72h-CGM

RCTs, randomized controlled trials; HbA1c, glycated hemoglobulin; T2DM, type 2 diabetes mellitus; MAGE, mean amplitude of glycemic excursions; R, randomized; DB, double blind; SB, single blind; OL, open-label; CO, crossover; Qd, once daily; Bid, twice daily; Tid, three times per day; CGM, continuous glucose monitoring; FGM, flash glucose monitoring.

**Table 2:** Details of study quality evaluation *via* the Cochrane’s Risk of Bias Tool.

Study	Randomsequencegeneration	Allocationconcealment	Blinding of participants	Blinding of outcome assessment	Incomplete outcome data addressed	Selectivereporting	Other sources of bias
(21)	Unclear	Unclear	High	High	Low	Low	Low
(22)	Unclear	Unclear	Low	Low	Low	Low	Low
(23)	Unclear	Unclear	Unclear	Unclear	Low	Low	Low
(24)	Unclear	Low	High	High	Low	Low	Low
(25)	Unclear	Unclear	Unclear	Unclear	Low	Low	Low
(29)	Unclear	Unclear	Unclear	Low	Low	Low	Low
(28-	Unclear	Low	High	Low	Low	Low	Low
(28)	Unclear	Low	High	Low	Low	Low	Low
(26)	Unclear	Unclear	High	High	Low	Low	Low
(27)	Unclear	Unclear	High	High	Low	Low	Low
(30)	Unclear	Unclear	High	High	Low	Low	Low
(31)	Unclear	Unclear	High	Low	Low	Low	Low
(32)	Unclear	Unclear	High	High	Low	Low	Low
(33)	Low	Unclear	High	Low	Low	Low	Low
(34)	Unclear	Unclear	High	Low	Low	Low	Low

### Comparisons between DPP4 inhibitors and other OADs on MAGE

**Figure 2:**
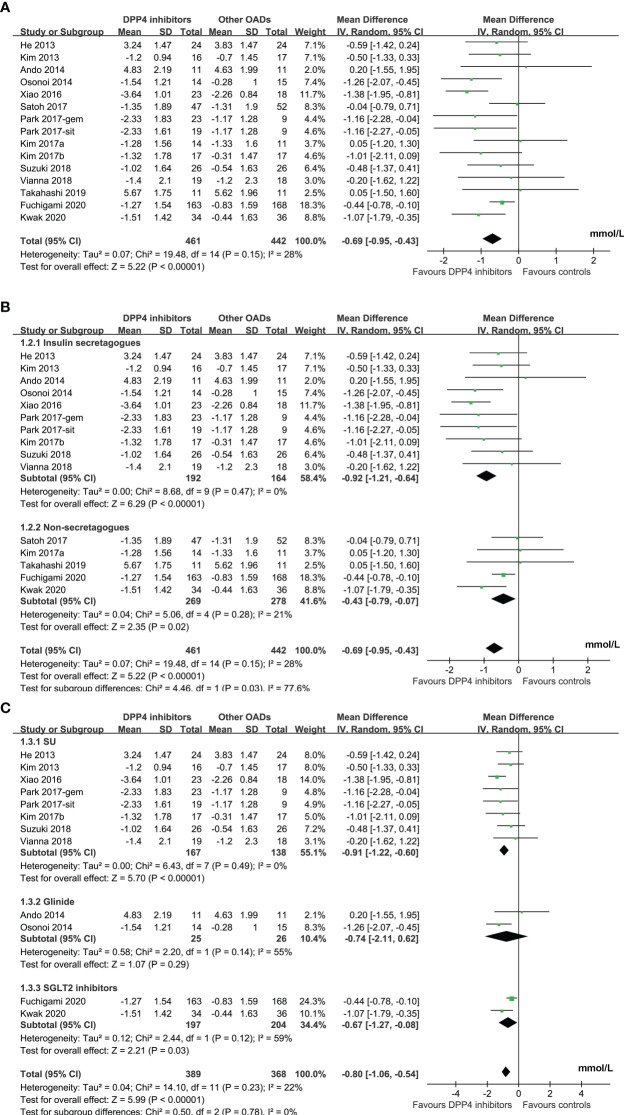
Forest plots for the meta-analysis comparing the influence of DPP4 inhibitors on MAGE with other OADs in T2DM patients with no concurrent insulin therapy; **(A)**, forest plots for the overall meta-analysis; **B)**, forest plots for the subgroup analysis according to the control OADs; and **(C)**, forest plots for the subgroup analysis according to the individual OAD class of controls.

Mild significant heterogeneity was detected among the included studies (P for Cochrane’s Q test = 0.15, I^2^ = 28%). Pooled results showed that DPP4 inhibitors significantly reduced MAGE compared to other OADs in T2DM patients with no concurrent insulin treatment (MD: -0.69 mmol/L, 95% CI: -0.95 to -0.43, P<0.001; **Figure 2A**). Sensitivity analysis by excluding one dataset at a time did not significantly change the results (MD: -0.57 to -0.75 mmol/L, P all < 0.05). Specifically, the results remained consistent after excluding the only study with FGM (MD: -0.75 mmol/L, 95% CI: -1.03 to -0.46, P<0.001; I^2^ = 23%). Predefined subgroup analyses suggested that DPP4 inhibitors were more effective at reducing MAGE compared to insulin secretagogues (MD: -0.92 mmol/L, 95% CI: -1.21 to -0.64, P < 0.001; I^2^ = 0%) and non-secretagogues (MD: -0.43 mmol/L, 95% CI: -0.79 to -0.07, P = 0.02; **Figure 2B**), as well as compared to sulfonylureas (MD: -0.91 mmol/L, 95% CI: -1.22 to -0.60, P < 0.001) and sodium glucose cotransporter 2 inhibitors ([SGLT-2] inhibitors, MD: -0.67 mmol/L, 95% CI: -1.27 to -0.08, P = 0.03, whereas the difference of MAGE between patients treated with DPP4 inhibitors and with glinides was not statistically significant (MD: -0.74 mmol/L, 95% CI: -2.11 to 0.62, P = 0.29; **Figure 2C**). In addition, subgroup analysis suggested that the reduction of MAGE was more remarkable in patients with T2DM duration ≤ 5 years as compared to those with disease duration > 5 years, and in studies with 72 hours of glucose monitoring for MAGE measurement as compared to those with 24 or 48 hours (P for subgroup difference = 0.004 and 0.002, respectively; **Table 3**). Other study characteristics did not seem to significantly affect the results, such as the study design, patient number, age and sex of the patients, HbA1c at baseline, background therapy, treatment duration, or quality scores of the included RCTs (**Table 3**). Subgroup analyses comparing DPP4 inhibitors to metformin or voglibose were not performed because only one dataset available for these comparisons.

**Table 3:** Subgroup analysis for comparing DPP4 inhibitors with other OADS on MAGE.

	Datasets	MD (95% CI)	P for subgroup effect	I^2^	P for subgroup difference
Design					
Crossover	3	-0.35 [-1.03, 0.33]	0.31	0%	
Parallel group	12	-0.74 [-1.04, -0.45]	< 0.001	38%	0.30
Patient number					
≤ 35	9	-0.74 [-1.09, -0.39]	< 0.001	0%	
> 35	6	-0.66 [-1.09, -0.23]	0.003	59%	0.78
Mean age (years)					
≤ 58	7	-0.63 [-1.06, -0.19]	0.005	0%	
> 58	8	-0.72 [-1.08, -0.36]	< 0.001	51%	0.74
Male (%)					
≤ 65	7	-0.57 [-0.98, -0.16]	0.006	51%	
> 65	7	-0.89 [-1.24, -0.54]	< 0.001	0%	0.25
Baseline HbA1c (%)					
≤ 7.5	7	-0.60 [-1.13, -0.07]	0.03	55%	
> 7.5	8	-0.62 [-0.87, -0.37]	< 0.001	0%	0.94
T2DM duration (years)					
≤ 5	5	-1.16 [-1.54, -0.79]	< 0.001	0%	
> 5	6	-0.48 [-0.75, -0.22]	< 0.001	0%	0.004
Background treatment					
Drug naïve	5	-0.52 [-1.02, -0.02]	0.04	16%	
With OAD	8	-0.80 [-1.19, -0.41]	< 0.001	57%	
Drug naïve or with OAD	2	-0.67 [-1.27, -0.08]	0.03	59%	0.69
Treatment duration (days)					
≤ 28	6	-0.62 [-1.02, -0.23]	0.002	0%	
> 28	9	-0.74 [-1.11, -0.37]	< 0.001	48%	0.68
CGM/FGM time (hours)					
24 or 48	8	-0.41 [-0.66, -0.15]	0.002	0%	
72	7	-1.05 [-1.37, -0.73]	< 0.001	5%	0.002
Quality score					
3	7	-0.67 [-1.14, -0.20]	0.005	34%	
4	4	-0.70 [-1.33, -0.07]	0.03	53%	
5	4	-0.55 [-0.84, -0.25]	< 0.001	0%	0.85

DPP4, dipeptidyl-peptidase 4; HbA1c, glycosylated hemoglobin; T2DM, type 2 diabetes mellitus; OAD, oral antidiabetic drug; MAGE, mean amplitude of glycemic excursions; MD, mean difference; CI, confidence interval; CGM, continuous glucose monitoring; FGM, flash glucose monitoring.

### Publication bias

**Figure 3:**
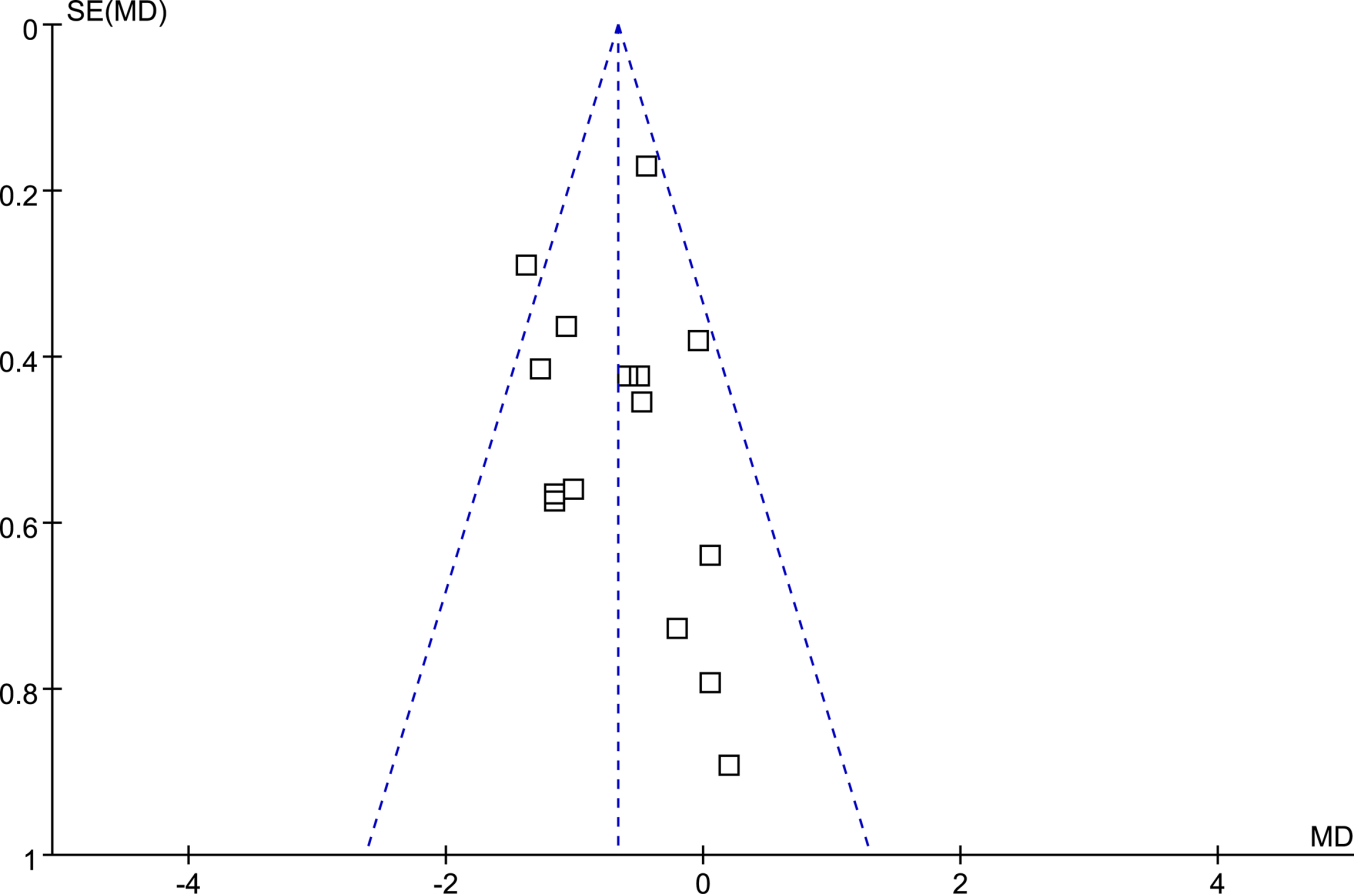
Funnel plots for the meta-analysis comparing the influence of DPP4 inhibitors on MAGE with other OADs in T2DM patients.

The funnel plots for the meta-analyses comparing the effects of DPP4 inhibitors and other OADs on MAGE were symmetrical, suggesting a low risk of publication bias (**Figure 3**). The results of Egger’s regression tests also suggested a low risk of publication bias (P = 0.301).

## Discussion

In this meta-analysis, by pooling the results of 14 RCTs, we found that DPP4 inhibitors were more effective than other OADs in attenuating glycemic variability in T2DM patients without concurrent insulin treatment, as evidenced by more significantly reduced MAGE after treatment with DPP4 inhibitors. The results of sensitivity analysis by excluding one dataset at a time showed consistent results, reflecting the stability of the findings. Interestingly, the results of subgroup analyses suggested that DPP4 inhibitors were more effective in reducing MAGE compared to insulin secretagogues and non-secretagogues, as well as compared to sulfonylureas and SGLT-2 inhibitors, in patients with T2DM duration ≤ 5 years as compared to those with disease duration > 5 years, and in studies with 72 hours of glucose monitoring for MAGE measurement as compared to those with 24 or 48 hours. Taken together, this meta-analysis confirmed that DPP4 inhibitors may significantly reduce glycemic variability as evidenced by MAGE in T2DM patients with no concurrent insulin treatment.

Currently, a number of parameters have been applied to evaluate the extent of glycemic variability. Among them, time in target range (TIR) is a new vital index of glycemic control that was recently proposed to describe the percentage of time when glucose values fall within the target range (the common default range of 3.9–10 mmol/L) in 24 h (40). Although this index is highly recommended by the American Diabetes Association (ADA) guidelines because TIR can not only be used to assess blood glucose fluctuation but also be used as a glycemic target besides HbA1c, studies using this parameter to evaluate the effect of anti-diabetic drugs on glycemic variability are still emerging (41). MAGE was considered as a gold standard for assessing the short-term within-day glycemic variability (GV), so that a large number of high-quality studies that used MAGEs for the evaluation of glucose fluctuation have been performed (42, 43). Previous studies showed that MAGE is independent of mean glycemic level (42) and associated with higher circulating insulin antibody (44), which may provide additional information for the evaluation and management of patients with T2DM (45). A cross-sectional study including 288 Chinese T2DM patients suggested that MAGE was better associated with vascular outcomes including coronary artery disease (CAD), stroke, and chronic kidney disease (CKD) than other indices of glycemic variability (46). Besides, previous studies showed that high MAGE was an independent predictive factor of poor prognosis in patients with CAD (9), including acute coronary syndrome (47, 48), and an early study showed that glycemic variability expressed as the MAGE was significantly associated with coronary plaque rupture in patients with acute myocardial infarction (AMI) (49). In addition, it was shown that high MAGE level was significantly associated with incidence of major adverse cardiovascular events after AMI (50). Similarly, in T2DM patients with acute ischemic stroke, MAGE was shown to be a predictor of early neurological deterioration (51). However, to the best of our knowledge, a clinically significant change margin of MAGE in patients with T2DM remains unknown, which needs more studies to investigate in the future. In addition, increased glycemic fluctuation is just one of the potential factors to the incidence of MACE in CVOTS. Nearly all CVOTs conducted with DPP4 inhibitors so far have demonstrated a neutral effect on MACE (52–56).

To the best of our knowledge, there is only one meta-analytic study that evaluated the influences of DPP4 inhibitors on glycemic variability compare to other OADs (35). This study included seven RCTs before 2018 and concluded that DPP4 inhibitors may be more effective than other OADs in reducing MAGE. The limited number of available datasets prevented the authors from performing further subgroup analyses. Moreover, the previous meta-analysis included a study with a methodological flaw in randomization (57), which significantly confounded the results of the meta-analysis. Compared to the previous meta-analysis, the strengths of the current meta-analysis include the rigorous literature search, strict inclusion and exclusion criteria, and performance of multiple sensitivity analysis to confirm the stability and robustness of the findings. We included 14 RCTs with 855 patients with T2DM, which was much larger than the previous meta-analysis (279 patients). The results suggested that DPP4 inhibitors are more effective than other OADs at attenuating glycemic variability in T2DM patients with no concurrent insulin therapy. This is of clinical importance. The available OADs for patients with T2DM have increased in recent decades. Although these OADs all have glucose-lowering efficacy, some may provide additional benefits for T2DM patients. In view of the potential role of increased glycemic variability in the pathogenesis of vascular complications in T2DM (12), agents that reduce glycemic variability may further improve the prognosis of T2DM patients.

The specific multidimensional mechanisms of DPP4 inhibitors for glycemic regulation, such as enhancing incretin preservation and inhibiting glucagon production (18, 19), may be the key reason that DPP4 inhibitors are more effective than other OADs in reducing MAGE. Further subgroup analysis according to individual OAD categories for the control showed that DPP4 inhibitors were more effective than SUs at reducing MAGE, whereas DPP4 inhibitors were comparable to glinides at reducing MAGE. This is consistent with previous observations that SUs are associated with higher glycemic fluctuation in T2DM patients (58). Moreover, glinides are short-acting insulin secretagogues and generally administered three times daily, which is also shown to be associated with reduced glucose fluctuation as compared with Sus (59). Interestingly, we also found that DPP4 inhibitors are more effective than SGLT-2 inhibitors in reducing MAGE. Of note, two of the included studies with SGLT-2 inhibitors as controls both used dapagliflozin. A recent experimental study in high-fat, high-sucrose diet mice showed that dapagliflozin increased plasma glucagon levels during acute declines in blood glucose (60), which may lead to increased glycemic fluctuation. Since only two RCTs were included in the subgroup of SGLT-2 inhibitors, further studies are needed to validate the findings and clarify the possible mechanisms underlying the superiority of DPP4 inhibitors to SGLT-2 inhibitors on MAGE.

Subgroup analysis also suggested that the reduction of MAGE was more remarkable in patients with T2DM duration ≤ 5 years as compared to those with disease duration > 5 years. Patients with longer T2DM duration are likely to have increased glycemic fluctuation severity, which is likely correlated with impaired pancreatic beta-cell function in these patients (61). Subsequently, the severe glycemic dysregulation in patients with long T2DM duration challenges hypoglycemic treatment to minimize glycemic fluctuations. In addition, a more remarkable reduction in MAGE after DPP4 inhibitor treatment was shown in studies with glucose monitoring for 72 hours as compared to those with 24 or 48-hour monitoring. These results indicate that the improvement of glycemic variability by DPP4 inhibitors was even more remarkable with a relatively longer monitoring duration.

This study also has limitations that should be considered when the results are interpreted. Firstly, there remains no consensus regarding the optimal parameter for measuring glycemic variability in T2DM patients. The influence of DPP4 inhibitors on other glycemic variability parameters in T2DM patients, such as TIR, should be evaluated. Besides, GV was analyzed based on CGM with relatively short durations among the included studies (24~72 hours). Parameters of GV calculated on the basis of CGM with longer duration (e.g., 14 days) may more accurately reflect the severity of glucose fluctuation. In addition, this meta-analysis was based on data at the study level rather than individual patients. Therefore, the results of subgroup analysis should be interpreted with caution. Large-scale RCTs or meta-analysis based on individual patient data may be considered to confirm whether patient characteristics or concurrent medications influence the potential effect of DPP4 inhibitors on MAGE. Finally, nine of the 14 RCTs included in the meta-analysis used insulin secretagogues (mostly SUs) as controls. None of the included studies compared DPP-4 inhibitors with the oral formulation of the GLP-1 receptor agonist semaglutide on GV. Studies are warranted for further investigation and the conclusion that DPP-4 inhibitors may be more effective than other OADs in reducing GV should be interpreted with caution in this regard.

## Conclusions

In conclusion, the results of the meta-analysis showed that DPP4 inhibitors may be more effective than other OADs in attenuating glycemic variability in T2DM patients receiving no concurrent insulin therapy.

## Data availability statement

The original contributions presented in the study are included in the article/supplementary material. Further inquiries can be directed to the corresponding author.

## Author contributions

SC, RZ, RC, YZ and MY conceived and designed research; SC and RZ collected data and conducted research; SC and MY performed or supervised analyses; SC, RZ, YMZ, SR and MY interpreted data; SC wrote the initial paper. All authors provided substantive suggestions for revision, reviewed and approved final version of the paper, and for all aspects of the work in ensuring that questions related to the accuracy.

## Funding

This study received funding from MSD China Holding Co. Ltd. for funding editorial assistance, which assistance was provided by Medjaden Inc.

## Acknowledgments

Jingya Chen of MSD China Holding Co., Ltd., Shanghai, China, assisted literature research of the manuscript. Administrative assistance was provided by Li Qi of MSD China Holding Co., Ltd., Shanghai, China. Medical writing and editorial assistance was provided by Medjaden Inc. This assistance was funded by MSD China.

## Conflict of interest

SC, RZ, YZ and YMZ are employees of MSD China; SR is employee of Merck Sharp & Dohme LLC., a subsidiary of Merck & Co., Inc., Rahway NJ, US. This study received funding from MSD China. The funder had the following involvement with the study: study design, data collection and analysis, and preparation of the manuscript. All authors declare no other competing interests.

The remaining authors declare that the research was conducted in the absence of any commercial or financial relationships that could be construed as a potential conflict of interest.

## Publisher’s note

All claims expressed in this article are solely those of the authors and do not necessarily represent those of their affiliated organizations, or those of the publisher, the editors and the reviewers. Any product that may be evaluated in this article, or claim that may be made by its manufacturer, is not guaranteed or endorsed by the publisher.

## Abbreviations

T2DM, type 2 diabetes mellitus; DPP4, dipeptidyl-peptidase 4; GLP-1, glucagon-like peptide-1; GIP, glucose-dependent insulinotropic polypeptide; FGM, flash glucose monitoring; RCT, randomized controlled trial; ADA, American Diabetes Association; CAD, coronary artery disease; CKD, chronic kidney disease; AMI, acute myocardial infarction; MACE, major adverse cardiovascular events; MAGE, mean amplitude of glycemic excursions.

## References

[1] Maranta F Cianfanelli L Cianflone D . Glycaemic control and vascular complications in diabetes mellitus type 2. Adv Exp Med Biol (2021) 1307:129–52. doi: 10.1007/5584_2020_514 32266607

[2] Zheng Y Ley SH Hu FB . Global aetiology and epidemiology of type 2 diabetes mellitus and its complications. Nat Rev Endocrinol (2018) 14:88–98. doi: 10.1038/nrendo.2017.151 29219149

[3] Buse JB Wexler DJ Tsapas A Rossing P Mingrone G Mathieu C . 2019 Update to: Management of hyperglycemia in type 2 diabetes, 2018. A consensus report by the American diabetes association (ADA) and the European association for the study of diabetes (EASD). Diabetes Care (2020) 43:487–93. doi: 10.2337/dci19-0066 PMC6971782 31857443

[4] Rodriguez-Gutierrez R Gonzalez-Gonzalez JG Zuniga-Hernandez JA McCoy RG . Benefits and harms of intensive glycemic control in patients with type 2 diabetes. BMJ (2019) 367:l5887. doi: 10.1136/bmj.l5887 31690574

[5] Rodbard D . Glucose variability: A review of clinical applications and research developments. Diabetes Technol Ther (2018) 20:S25–S215. doi: 10.1089/dia.2018.0092 29916742

[6] Nusca A Tuccinardi D Albano M Cavallaro C Ricottini E Manfrini S . Glycemic variability in the development of cardiovascular complications in diabetes. Diabetes Metab Res Rev (2018) 34:e3047. doi: 10.1002/dmrr.3047 30028067

[7] Kovatchev B . Glycemic variability: Risk factors, assessment, and control. J Diabetes Sci Technol (2019) 13:627–35. doi: 10.1177/1932296819826111 PMC6610616 30694076

[8] Gorst C Kwok CS Aslam S Buchan I Kontopantelis E Myint PK . Long-term glycemic variability and risk of adverse outcomes: A systematic review and meta-analysis. Diabetes Care (2015) 38:2354–69. doi: 10.2337/dc15-1188 26604281

[9] Pu Z Lai L Yang X Wang Y Dong P Wang D . Acute glycemic variability on admission predicts the prognosis in hospitalized patients with coronary artery disease: a meta-analysis. Endocrine (2020) 67:526–34. doi: 10.1007/s12020-019-02150-1 31828526

[10] Nalysnyk L Hernandez-Medina M Krishnarajah G . Glycaemic variability and complications in patients with diabetes mellitus: evidence from a systematic review of the literature. Diabetes Obes Metab (2010) 12:288–98. doi: 10.1111/j.1463-1326.2009.01160.x 20380649

[11] Monnier L Mas E Ginet C Michel F Villon L Cristol JP . Activation of oxidative stress by acute glucose fluctuations compared with sustained chronic hyperglycemia in patients with type 2 diabetes. JAMA (2006) 295:1681–7. doi: 10.1001/jama.295.14.1681 16609090

[12] Valente T Arbex AK . Glycemic variability, oxidative stress and impact on complications related to type 2 diabetes mellitus. Curr Diabetes Rev (2021) 17(7):e071620183816. doi: 10.2174/1573399816666200716201550 32674737

[13] van Genugten RE van Raalte DH Diamant M . Dipeptidyl peptidase-4 inhibitors and preservation of pancreatic islet-cell function: a critical appraisal of the evidence. Diabetes Obes Metab (2012) 14:101–11. doi: 10.1111/j.1463-1326.2011.01473.x 21752172

[14] Chen K Kang D Yu M Zhang R Zhang Y Chen G . Direct head-to-head comparison of glycaemic durability of dipeptidyl peptidase-4 inhibitors and sulphonylureas in patients with type 2 diabetes mellitus: A meta-analysis of long-term randomized controlled trials. Diabetes Obes Metab (2018) 20:1029–33. doi: 10.1111/dom.13147 PMC5873267 29095568

[15] Lefebvre PJ Paquot N Scheen AJ . Inhibiting or antagonizing glucagon: making progress in diabetes care. Diabetes Obes Metab (2015) 17:720–5. doi: 10.1111/dom.12480 25924114

[16] Kohnert KD Freyse EJ Salzsieder E . Glycaemic variability and pancreatic beta-cell dysfunction. Curr Diabetes Rev (2012) 8:345–54. doi: 10.2174/157339912802083513 22698079

[17] Rizzo MR Barbieri M Marfella R Paolisso G . Reduction of oxidative stress and inflammation by blunting daily acute glucose fluctuations in patients with type 2 diabetes: role of dipeptidyl peptidase-IV inhibition. Diabetes Care (2012) 35:2076–82. doi: 10.2337/dc12-0199 PMC3447848 22688551

[18] Mulvihill EE Drucker DJ . Pharmacology, physiology, and mechanisms of action of dipeptidyl peptidase-4 inhibitors. Endocr Rev (2014) 35:992–1019. doi: 10.1210/er.2014-1035 25216328 PMC7108477

[19] Thornberry NA Gallwitz B . Mechanism of action of inhibitors of dipeptidyl-peptidase-4 (DPP-4). Best Pract Res Clin Endocrinol Metab (2009) 23:479–86. doi: 10.1016/j.beem.2009.03.004 19748065

[20] Ajjan RA Cummings MH Jennings P Leelarathna L Rayman G Wilmot EG . Accuracy of flash glucose monitoring and continuous glucose monitoring technologies: Implications for clinical practice. Diabetes Vasc Dis Res (2018) 15:175–84. doi: 10.1177/1479164118756240 29446646

[21] He YL Foteinos G Neelakantham S Mattapalli D Kulmatycki K Forst T . Differential effects of vildagliptin and glimepiride on glucose fluctuations in patients with type 2 diabetes mellitus assessed using continuous glucose monitoring. Diabetes Obes Metab (2013) 15:1111–9. doi: 10.1111/dom.12146 23782529

[22] Kim HS Shin JA Lee SH Kim ES Cho JH Son HY . A comparative study of the effects of a dipeptidyl peptidase-IV inhibitor and sulfonylurea on glucose variability in patients with type 2 diabetes with inadequate glycemic control on metformin. Diabetes Technol Ther (2013) 15:810–6. doi: 10.1089/dia.2013.0038 24050737

[23] Ando K Nishimura R Seo C Tsujino D Sakamoto M Utsunomiya K . Comparing postprandial efficacy in type 2 diabetic patients receiving mitiglinide and sitagliptin by using continuous glucose monitoring: a pilot study. Expert Opin Pharmacother (2014) 15:2479–85. doi: 10.1517/14656566.2014.970531 25327311

[24] Osonoi T Saito M Tamasawa A Ishida H Osonoi Y . Effects of sitagliptin or mitiglinide as an add-on to acarbose on daily blood glucose fluctuations measured by 72 h subcutaneous continuous glucose monitoring in Japanese patients with type 2 diabetes: a prospective randomized study. Expert Opin Pharmacother (2014) 15:1325–35. doi: 10.1517/14656566.2014.920323 24866329

[25] Xiao X Cui X Zhang J Han Z Xiao Y Chen N . Effects of sitagliptin as initial therapy in newly diagnosed elderly type 2 diabetics: A randomized controlled study. Exp Ther Med (2016) 12:3002–8. doi: 10.3892/etm.2016.3729 PMC5103735 27882107

[26] Kim G Oh S Jin SM Hur KY Kim JH Lee MK . The efficacy and safety of adding either vildagliptin or glimepiride to ongoing metformin therapy in patients with type 2 diabetes mellitus. Expert Opin Pharmacother (2017) 18:1179–86. doi: 10.1080/14656566.2017.1353080 28714741

[27] Kim NH Kim DL Kim KJ Choi KM Baik SH Kim SG . Effects of vildagliptin or pioglitazone on glycemic variability and oxidative stress in patients with type 2 diabetes inadequately controlled with metformin monotherapy: A 16-week, randomised, open label, pilot study. Endocrinol Metab (Seoul) (2017) 32:241–7. doi: 10.3803/EnM.2017.32.2.241 PMC5503869 28685513

[28] Park SE Lee BW Kim JH Lee WJ Cho JH Jung CH . Effect of gemigliptin on glycaemic variability in patients with type 2 diabetes (STABLE study). Diabetes Obes Metab (2017) 19:892–6. doi: 10.1111/dom.12869 28058753

[29] Satoh H Ohira T Moriya C Inoue I Kuribayashi S Seino H . Effects of linagliptin vs. voglibose on daily glucose excursions during continuous glucose monitoring of Japanese type 2 diabetes patients (L-CGM): A randomized, open-label, two-arm, parallel comparative trial. Diabetes Metab (2017) 43:550–3. doi: 10.1016/j.diabet.2017.07.010 28947255

[30] Suzuki R Eiki JI Moritoyo T Furihata K Wakana A Ohta Y . Effect of short-term treatment with sitagliptin or glibenclamide on daily glucose fluctuation in drug-naive Japanese patients with type 2 diabetes mellitus. Diabetes Obes Metab (2018) 20:2274–81. doi: 10.1111/dom.13364 29770541

[31] Vianna AGD Lacerda CS Pechmann LM Polesel MG Marino EC Faria-Neto JR . A randomized controlled trial to compare the effects of sulphonylurea gliclazide MR (modified release) and the DPP-4 inhibitor vildagliptin on glycemic variability and control measured by continuous glucose monitoring (CGM) in Brazilian women with type 2 diabetes. Diabetes Res Clin Pract (2018) 139:357–65. doi: 10.1016/j.diabres.2018.03.035 29596951

[32] Takahashi H Nishimura R Tsujino D Utsunomiya K . Which is better, high-dose metformin monotherapy or low-dose metformin/linagliptin combination therapy, in improving glycemic variability in type 2 diabetes patients with insufficient glycemic control despite low-dose metformin monotherapy? a randomized, cross-over, continuous glucose monitoring-based pilot study. J Diabetes Investig (2019) 10:714–22. doi: 10.1111/jdi.12922 PMC6497608 30171747

[33] Fuchigami A Shigiyama F Kitazawa T Okada Y Ichijo T Higa M . Efficacy of dapagliflozin versus sitagliptin on cardiometabolic risk factors in Japanese patients with type 2 diabetes: a prospective, randomized study (DIVERSITY-CVR). Cardiovasc Diabetol (2020) 19:1. doi: 10.1186/s12933-019-0977-z 31910850 PMC6945792

[34] Kwak SH Hwang YC Won JC Bae JC Kim HJ Suh S . Comparison of the effects of gemigliptin and dapagliflozin on glycaemic variability in type 2 diabetes: A randomized, open-label, active-controlled, 12-week study (STABLE II study). Diabetes Obes Metab (2020) 22:173–81. doi: 10.1111/dom.13882 31502749

[35] Lee S Lee H Kim Y Kim E . Effect of DPP-IV inhibitors on glycemic variability in patients with T2DM: A systematic review and meta-analysis. Sci Rep (2019) 9:13296. doi: 10.1038/s41598-019-49803-9 31527625 PMC6746852

[36] Moher D Liberati A Tetzlaff J Altman DG . Preferred reporting items for systematic reviews and meta-analyses: the PRISMA statement. BMJ (2009) 339:b2535. doi: 10.1136/bmj.b2535 19622551 PMC2714657

[37] Higgins J Green S . Cochrane handbook for systematic reviews of interventions version 5.1.0. The Cochrane Collaboration London (2011). Available at: www.cochranehandbook.org.

[38] Higgins JP Thompson SG . Quantifying heterogeneity in a meta-analysis. Stat Med (2002) 21:1539–58. doi: 10.1002/sim.1186 12111919

[39] Egger M Davey Smith G Schneider M Minder C . Bias in meta-analysis detected by a simple, graphical test. BMJ (1997) 315:629–34. doi: 10.1136/bmj.315.7109.629 PMC2127453 9310563

[40] Yoo JH Kim JH . Time in range from continuous glucose monitoring: A novel metric for glycemic control. Diabetes Metab J (2020) 44:828–39. doi: 10.4093/dmj.2020.0257 PMC7801761 33389957

[41] Gabbay MAL Rodacki M Calliari LE Vianna AGD Krakauer M Pinto MS . Time in range: a new parameter to evaluate blood glucose control in patients with diabetes. Diabetol Metab Syndr (2020) 12:22. doi: 10.1186/s13098-020-00529-z 32190124 PMC7076978

[42] Monnier L Colette C Owens DR . The application of simple metrics in the assessment of glycaemic variability. Diabetes Metab (2018) 44:313–9. doi: 10.1016/j.diabet.2018.02.008 29602622

[43] Verges B Pignol E Rouland A Bouillet B Baillot-Rudoni S Quilot E . Glycemic variability assessment with a 14-day continuous glucose monitoring system: When and how long to measure MAGE (Mean amplitude of glucose excursion) for optimal reliability? J Diabetes Sci Technol (2022) 16(4):982–7. doi: 10.1177/1932296821992060 PMC9264451 33567877

[44] Zhu J Yuan L Ni WJ Luo Y Ma JH . Association of higher circulating insulin antibody with increased mean amplitude glycemic excursion in patients with type 2 diabetes mellitus: A cross-sectional, retrospective case-control study. J Diabetes Res (2019) 2019:7304140. doi: 10.1155/2019/7304140 31687408 PMC6800966

[45] Monnier L Colette C Owens DR . Integrating glycaemic variability in the glycaemic disorders of type 2 diabetes: a move towards a unified glucose tetrad concept. Diabetes Metab Res Rev (2009) 25:393–402. doi: 10.1002/dmrr.962 19437415

[46] Tong L Chi C Zhang Z . Association of various glycemic variability indices and vascular outcomes in type-2 diabetes patients: A retrospective study. Med (Baltimore) (2018) 97:e10860. doi: 10.1097/MD.0000000000010860 PMC6392700 29794785

[47] Takahashi H Iwahashi N Kirigaya J Kataoka S Minamimoto Y Gohbara M . Glycemic variability determined with a continuous glucose monitoring system can predict prognosis after acute coronary syndrome. Cardiovasc Diabetol (2018) 17:116. doi: 10.1186/s12933-018-0761-5 30121076 PMC6098663

[48] Su G Zhang T Yang H Dai W Tian L Tao H . Admission glycemic variability correlates with in-hospital outcomes in diabetic patients with non-ST segment elevation acute coronary syndrome undergoing percutaneous coronary intervention. Anatol J Cardiol (2018) 19:368–73. doi: 10.14744/AnatolJCardiol.2018.47487 PMC5998864 29848920

[49] Teraguchi I Imanishi T Ozaki Y Tanimoto T Orii M Shiono Y . Impact of glucose fluctuation and monocyte subsets on coronary plaque rupture. Nutr Metab Cardiovasc Dis (2014) 24:309–14. doi: 10.1016/j.numecd.2013.08.010 24418379

[50] Su G Mi SH Tao H Li Z Yang HX Zheng H . Impact of admission glycemic variability, glucose, and glycosylated hemoglobin on major adverse cardiac events after acute myocardial infarction. Diabetes Care (2013) 36:1026–32. doi: 10.2337/dc12-0925 PMC3609497 23349547

[51] Hui J Zhang J Mao X Li Z Li X Wang F . The initial glycemic variability is associated with early neurological deterioration in diabetic patients with acute ischemic stroke. Neurol Sci (2018) 39:1571–7. doi: 10.1007/s10072-018-3463-6 29869743

[52] White WB Cannon CP Heller SR Nissen SE Bergenstal RM Bakris GL . Alogliptin after acute coronary syndrome in patients with type 2 diabetes. N Engl J Med (2013) 369:1327–35. doi: 10.1056/NEJMoa1305889 23992602

[53] Scirica BM Bhatt DL Braunwald E Steg PG Davidson J Hirshberg B . Saxagliptin and cardiovascular outcomes in patients with type 2 diabetes mellitus. N Engl J Med (2013) 369:1317–26. doi: 10.1056/NEJMoa1307684 23992601

[54] Green JB Bethel MA Armstrong PW Buse JB Engel SS Garg J . Effect of sitagliptin on cardiovascular outcomes in type 2 diabetes. N Engl J Med (2015) 373:232–42. doi: 10.1056/NEJMoa1501352 26052984

[55] Rosenstock J Perkovic V Johansen OE Cooper ME Kahn SE Marx N . Effect of linagliptin vs placebo on major cardiovascular events in adults with type 2 diabetes and high cardiovascular and renal risk: The CARMELINA randomized clinical trial. JAMA (2019) 321:69–79. doi: 10.1001/jama.2018.18269 30418475 PMC6583576

[56] Rosenstock J Kahn SE Johansen OE Zinman B Espeland MA Woerle HJ . Effect of linagliptin vs glimepiride on major adverse cardiovascular outcomes in patients with type 2 diabetes: The CAROLINA randomized clinical trial. Jama (2019) 322:1155–66. doi: 10.1001/jama.2019.13772 PMC6763993 31536101

[57] Park KS Kwak S Cho YM Jang HC Kim SY Jung HS . Vildagliptin reduces plasma stromal cell-derived factor-1alpha in patients with type 2 diabetes compared with glimepiride. J Diabetes Investig (2017) 8:218–26. doi: 10.1111/jdi.12572 PMC5334315 27575011

[58] Yoo S Chin SO Lee SA Koh G . Factors associated with glycemic variability in patients with type 2 diabetes: Focus on oral hypoglycemic agents and cardiovascular risk factors. Endocrinol Metab (Seoul) (2015) 30:352–60. doi: 10.3803/EnM.2015.30.3.352 PMC4595361 26248860

[59] Omori K Nomoto H Nakamura A Takase T Cho KY Ono K . Reduction in glucose fluctuations in elderly patients with type 2 diabetes using repaglinide: A randomized controlled trial of repaglinide vs sulfonylurea. J Diabetes Investig (2019) 10:367–74. doi: 10.1111/jdi.12889 PMC6400204 29963781

[60] Suga T Kikuchi O Kobayashi M Matsui S Yokota-Hashimoto H Wada E . SGLT1 in pancreatic alpha cells regulates glucagon secretion in mice, possibly explaining the distinct effects of SGLT2 inhibitors on plasma glucagon levels. Mol Metab (2019) 19:1–12. doi: 10.1016/j.molmet.2018.10.009 30416006 PMC6323192

[61] Takai M Anno T Kawasaki F Kimura T Hirukawa H Mune T . Association of the glycemic fluctuation as well as glycemic control with the pancreatic beta-cell function in Japanese subjects with type 2 diabetes mellitus. Intern Med (2019) 58:167–73. doi: 10.2169/internalmedicine.1053-18 PMC6378157 30146574

